# Supplementary material for: The Green Tea Polyphenol Epigallocatechin-Gallate (EGCG) Interferes with Microcin E492 Amyloid Formation
Source: Molecules. 2023 Oct 25;28(21):7262. doi: 10.3390/molecules28217262 (PMC10648153; doi:10.3390/molecules28217262)
Supplement: Supplementary file 1 [file molecules-28-07262-s001.zip › molecules-2652228-supplementary.pdf]

The green tea polyphenol Epigallocatechin-Gallate (EGCG) interferes with microcin E492 amyloid formation

Paulina Aguilera[1], Camilo Berríos-Pastén[1], Marcelo Veloso[1], Matías Gálvez-Silva[1], Florian Turbant[2,3], Rosalba Lagos[1], Frank Wien[2], Veronique Arluison[3,4,*] and Andrés E. Marcoleta[1,*]

[1]Grupo de Microbiología Integrativa, Laboratorio de Biología Estructural y Molecular BEM, Facultad de Ciencias, Universidad de Chile. Las Palmeras 3425 Ñuñoa, Santiago, Chile.

[2]Synchrotron SOLEIL, L'Orme des Merisiers, Saint Aubin BP48, 91192 Gif-sur-Yvette, France.

[3]Laboratoire Léon Brillouin LLB, CEA, CNRS UMR12, Université Paris-Saclay, CEA Saclay, 91191 Gif-sur-Yvette, France.

[4]Université Paris Cité, UFR Sciences du vivant, 75006 Paris, France.

*Correspondence: VA: veronique.arluison@u-paris.fr, AM: amarcoleta@uchile.cl

SUPPLEMENTARY MATERIAL

Contents:

Figure S1. Transmission electron microscopy visualization of MccE492 fibers in presence and absence of EGCG.

Figure S2. MccE492 secondary structure content at different temperatures in the absence or presence of EGCG determined using BestSel.

Figure S3. MccE492 antibacterial activity in the presence or absence of EGCG.

Figure S4. Acetonitrile (ACN)-gradient fractions obtained after a typical MccE492 purification.

Table S1. Secondary structure content of MccE492 amyloid at different temperatures in the absence of 1 mM EGCG.

Table S2. Secondary structure content of MccE492 amyloid at different temperatures in 1 mM EGCG.

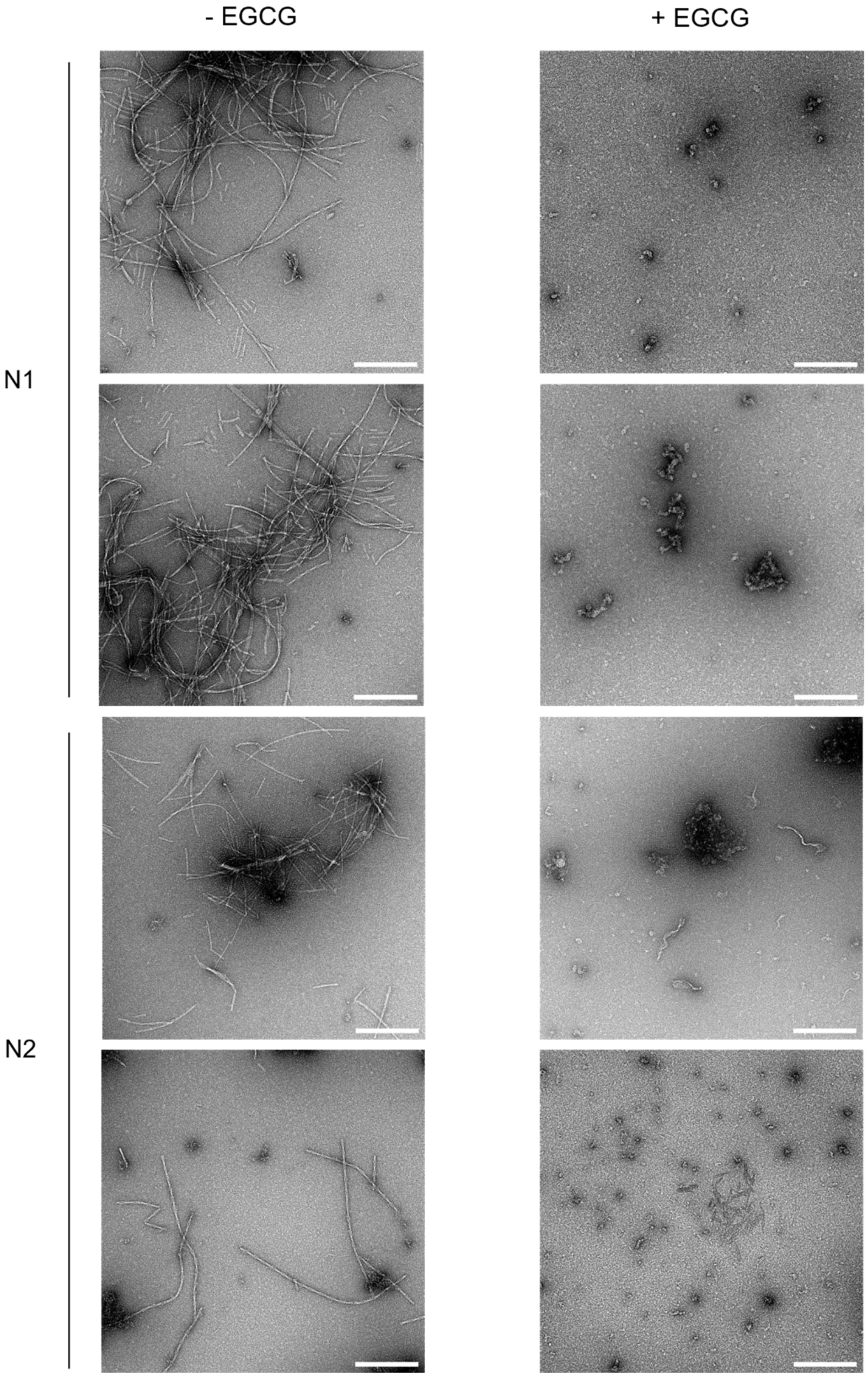

Figure S1. Visualization of MccE492 fibers in presence and absence of EGCG. Several fields of negative-stain electron microscopy images show the effect of EGCG in the formation of amyloid fibers after 48 h in PIPES aggregation buffer. These images correspond to two independent experiments (N1 and N2). Scale bar: 200 nm.

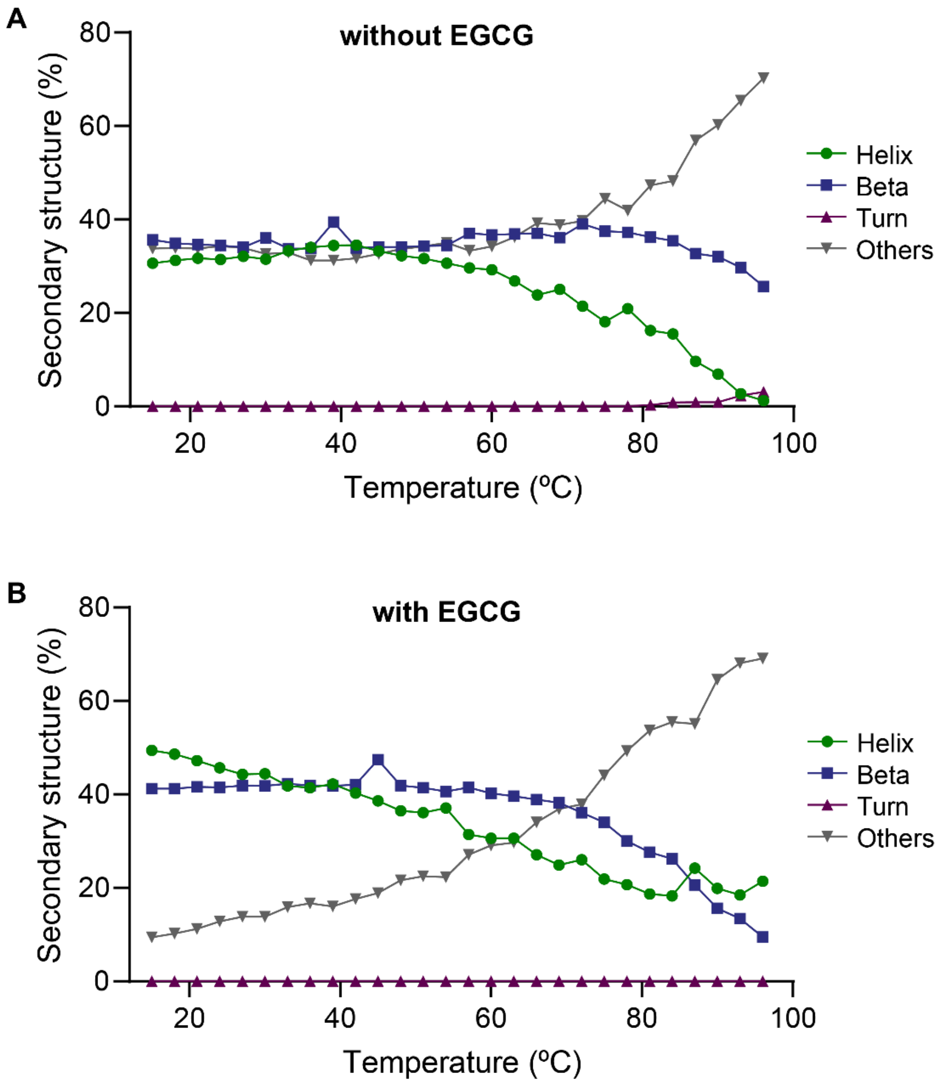

Figure S2. MccE492 secondary structure content at different temperatures in the absence (A) or presence (B) of EGCG determined using the BestSel software.

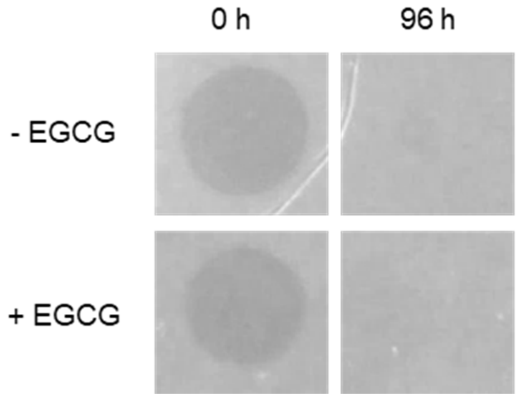

Figure S3. MccE492 antibacterial activity in the presence or absence of EGCG. Three μL of the samples from the MccE492 aggregation assays in PIPES buffer were seeded onto a lawn of sensitive *E. coli* strain. The formation of growth inhibition halos denotes MccE492 antibacterial activity.

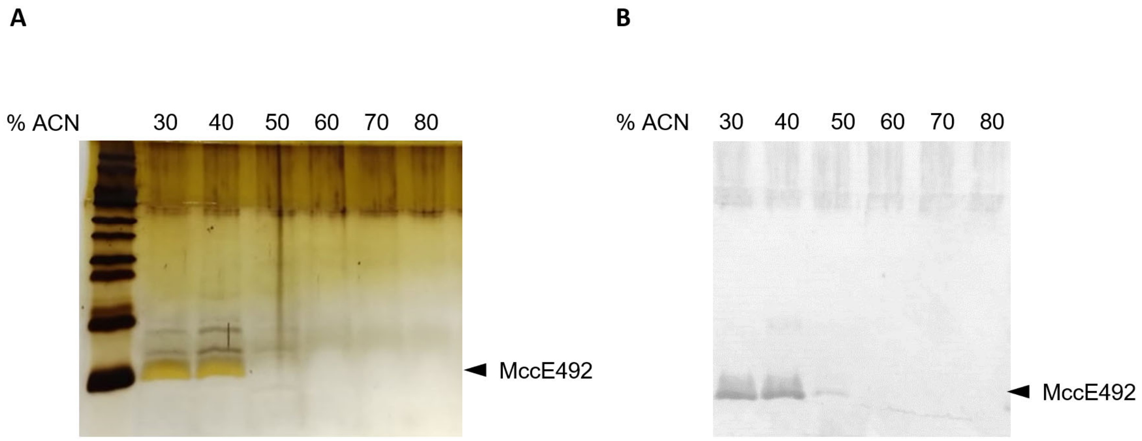

Figure S4. Acetonitrile (ACN)-gradient fractions obtained after a typical MccE492 purification. The ACN fractions recovered after the purifications were checked by SDS-PAGE and silver-staining **(A)**, and Western blot **(B).**

Table S1. Secondary structure content of MccE492 amyloid at different temperatures in the absence of 1 mM EGCG.

Temperature (°C)	Helix		β-strand				Turn	Others	NRMSD
	Helix1	Helix2	Anti1	Anti2	Anti3	Para			
**15**	19.1	11.5	0.0	0.0	0.0	35.6	0.0	33.8	0.06929
**18**	18.8	12.4	0.0	0.0	0.0	34.9	0.0	33.9	0.07098
**21**	19.1	12.6	0.0	0.0	0.0	34.6	0.0	33.8	0.07102
**24**	19.1	12.3	0.0	0.0	0.0	34.4	0.0	34.3	0.07417
**27**	19.3	12.8	0.0	0.0	0.0	34.0	0.0	33.9	0.07579
**30**	18.4	13.1	0.0	0.0	2.2	33.8	0.0	32.6	0.07670
**33**	19.6	13.7	0.0	0.0	0.0	33.8	0.0	32.9	0.07399
**36**	20.0	14.0	0.0	0.0	0.0	33.8	0.0	32.2	0.07806
**39**	20.5	13.9	0.0	0.0	2.5	31.9	0.0	31.2	0.07726
**42**	20.6	13.9	0.0	0.0	2.0	31.9	0.0	31.7	0.07382
**45**	19.5	13.7	0.0	0.0	0.9	33.2	0.0	32.6	0.07647
**48**	18.3	13.7	0.0	0.0	0.4	33.7	0.0	33.7	0.07241
**51**	18.2	13.4	0.0	0.0	1.0	33.3	0.0	34.2	0.07667
**54**	17.3	13.3	0.0	0.0	2.4	32.0	0.0	35.0	0.07372
**57**	16.4	13.2	0.0	0.0	4.9	32.1	0.0	33.3	0.06603
**60**	16.0	13.2	0.0	0.0	4.5	32.2	0.0	34.2	0.06598
**63**	14.4	12.4	0.0	0.0	1.8	35.1	0.0	36.2	0.06676
**66**	12.4	11.4	0.0	0.0	3.4	33.6	0.0	39.2	0.06645
**69**	12.9	12.1	0.0	0.0	2.2	33.9	0.0	38.8	0.06910
**72**	11.7	9.7	0.0	0.0	4.2	34.8	0.0	39.7	0.06416
**75**	9.8	8.3	0.0	0.0	3.2	34.3	0.0	44.4	0.06882
**78**	10.5	10.4	0.0	0.0	3.7	33.5	0.0	41.9	0.06850
**81**	7.8	8.4	0.0	0.0	2.5	33.7	0.3	47.3	0.06921
**84**	7.1	8.4	0.0	0.0	3.1	32.3	0.8	48.2	0.07211
**87**	3.8	5.8	0.0	0.0	0.0	32.7	0.9	56.9	0.07820
**90**	2.3	4.6	0.0	0.0	3.4	28.6	0.9	60.2	0.08155
**93**	0.3	2.4	0.0	0.0	1.5	28.2	2.3	65.4	0.07661
**96**	0.0	1.2	0.0	0.0	0.0	25.6	3.1	70.2	0.09442

Table S2. Secondary structure content of MccE492 amyloid at different temperatures in the presence of 1 mM EGCG.

Temperature (°C)	Helix		β-strand				Turn	Others	NRMSD
	Helix1	Helix2	Anti1	Anti2	Anti3	Para			
**15**	39.0	10.4	0.0	0.0	0.0	41.2	0.0	9.4	0.09809
**18**	36.7	11.9	0.0	0.0	0.0	41.2	0.0	10.2	0.09928
**21**	35.4	11.8	0.0	0.0	0.0	41.6	0.0	11.2	0.09880
**24**	34.2	11.5	0.0	0.0	0.0	41.5	0.0	12.8	0.10016
**27**	32.0	12.3	0.0	0.0	0.0	41.9	0.0	13.8	0.09680
**30**	32.8	11.6	0.0	0.0	0.0	41.8	0.0	13.8	0.10406
**33**	30.1	11.7	0.0	0.0	0.0	42.3	0.0	15.9	0.10098
**36**	29.3	12.1	0.0	0.0	0.0	41.9	0.0	16.7	0.10351
**39**	30.7	11.5	0.0	0.0	0.0	41.8	0.0	16.0	0.10203
**42**	28.6	11.7	0.0	0.0	0.0	42.1	0.0	17.6	0.10003
**45**	27.0	11.6	0.0	0.0	0.0	42.4	0.0	18.9	0.09940
**48**	24.8	11.7	0.0	0.0	0.0	41.9	0.0	21.6	0.09985
**51**	24.7	11.4	0.0	0.0	0.0	41.4	0.0	22.5	0.09649
**54**	26.3	10.8	0.0	0.0	0.0	40.6	0.0	22.3	0.09696
**57**	21.4	10.0	0.0	0.0	0.0	41.5	0.0	27.1	0.09747
**60**	21.0	9.6	0.0	0.0	0.0	40.2	0.0	29.1	0.09845
**63**	21.7	8.9	0.0	0.0	0.0	39.6	0.0	29.7	0.09542
**66**	20.1	7.0	0.0	0.0	0.0	38.9	0.0	34.0	0.10341
**69**	19.0	5.9	0.0	0.0	0.0	38.2	0.0	36.9	0.10988
**72**	21.5	4.5	0.0	0.0	0.0	36.1	0.0	37.9	0.10791
**75**	18.4	3.5	0.0	0.0	0.0	34.0	0.0	44.1	0.11015
**78**	19.7	1.0	0.0	0.0	0.0	30.0	0.0	49.3	0.11028
**81**	18.7	0.0	0.0	0.0	0.0	27.6	0.0	53.7	0.11804
**84**	18.3	0.0	0.0	0.0	0.0	26.2	0.0	55.5	0.12599
**87**	24.2	0.0	0.0	0.0	0.0	20.6	0.0	55.1	0.11457
**90**	19.9	0.0	0.0	0.0	0.0	15.6	0.0	64.6	0.12205
**93**	18.5	0.0	0.0	0.0	0.0	13.4	0.0	68.1	0.12198
**96**	21.4	0.0	0.0	0.0	0.0	9.5	0.0	69.1	0.12917
